# Systemic Necrotizing Medium-Vessel Vasculitis Clinically Consistent With Polyarteritis Nodosa: A Detailed Case Report

**DOI:** 10.7759/cureus.102178

**Published:** 2026-01-23

**Authors:** Niyas Khalid Ottu Para, Seema Rab, Asiya Pathan, Eman F Sharaf

**Affiliations:** 1 Internal Medicine, Burjeel Hospital Abu Dhabi, Abu Dhabi, ARE; 2 Internal Medicine, Burjeel Holdings, Abu Dhabi, ARE

**Keywords:** anca-negative vasculitis, cutaneous vasculitis, medium-vessel vasculitis, mononeuritis multiplex, necrotizing vasculitis, polyarteritis nodosa

## Abstract

Polyarteritis nodosa (PAN) is a rare necrotizing vasculitis of medium-sized arteries presenting with heterogeneous multisystem involvement. Diagnosis is challenging due to the absence of specific biomarkers, frequent anti-neutrophil cytoplasmic antibodies (ANCA) negativity, variable early imaging findings, and low diagnostic yield of superficial biopsies. We report the case of a 33-year-old man experiencing a four-month evolution of recurrent inflammatory episodes involving abdominal, neurological, cutaneous, and cardiopulmonary systems. He developed dusky necrotizing vasculitic lesions, mononeuritis multiplex-type neuropathy, constitutional symptoms, markedly elevated inflammatory markers, and demonstrated a dramatic response to corticosteroid therapy. Computed tomography angiography was normal, and a superficial punch biopsy performed after steroid initiation was nondiagnostic, reflecting known limitations of sampling depth and timing. Despite this, clinical features, disease trajectory, exclusion of alternative vasculitides, and classical steroid responsiveness strongly supported systemic PAN. This case highlights the diagnostic limitations of biopsy and imaging in early PAN and emphasizes the need for clinical judgment and timely treatment.

## Introduction

Polyarteritis nodosa (PAN) is an uncommon systemic necrotizing vasculitis characterized by inflammation of medium-sized muscular arteries, typically sparing arterioles, capillaries, and venules. Its estimated annual incidence ranges from two to nine cases per million [1]. Historically, PAN encompassed several subtypes of vasculitis, but the 2012 Revised International Chapel Hill Consensus Conference (CHCC) reclassified PAN strictly as a medium-vessel vasculitis and distinguished it from microscopic polyangiitis (MPA) and other small-vessel vasculitides [2]. A key feature of PAN is the absence of glomerulonephritis, pulmonary capillaritis, or ear-nose-throat (ENT) involvement, features commonly associated with anti-neutrophil cytoplasmic antibodies (ANCA)-associated vasculitis (AAV) [2,3]. ANCA is typically negative in PAN, which helps differentiate it from granulomatosis with polyangiitis (GPA), eosinophilic GPA (EGPA), and MPA [3,4]. Unlike immune-complex vasculitides, complement levels in PAN are usually normal or slightly elevated as part of the acute-phase response [5]. Diagnosis of PAN remains difficult because there is no single biomarker or imaging test that is definitively diagnostic. Angiographic sensitivity is limited in early disease because aneurysms and stenoses may take months to develop. Biopsy sensitivity depends heavily on sampling depth, as medium-sized arteries are located in the deep dermis and subcutis, so superficial punch biopsies often miss the diagnostic vessels [6,7]. Furthermore, corticosteroids rapidly suppress inflammation, reducing biopsy yield within 24-48 hours [6]. For these reasons, PAN remains, in many cases, a clinical diagnosis based on the pattern of organ involvement, characteristic neuropathy and skin lesions, inflammatory markers, exclusion of mimics like GPA, EGPA, and MPA, and therapeutic response to immunosuppression. We present a diagnostically complex case of systemic PAN in a young man with recurrent inflammatory episodes, vasculitic neuropathy, and necrotic skin lesions in whom early imaging and superficial biopsy were nondiagnostic. The case illustrates the limitations of conventional diagnostics and underscores the central role of clinical judgment and early treatment.

## Case presentation

**Figure 1:**
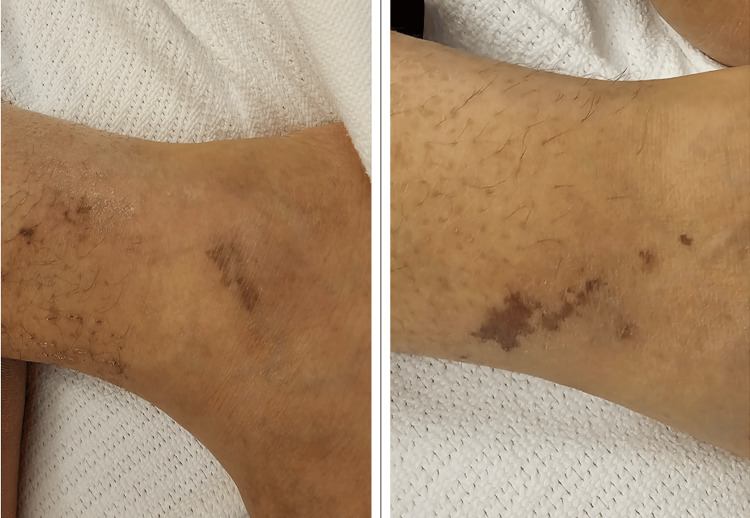
Cutaneous Manifestations of Polyarteritis Nodosa (PAN). Dusky violaceous rashes involving the ankle, representing cutaneous manifestations of PAN. The lesions lacked features of any active infection, prompting evaluation for systemic vasculitis.

A 33-year-old previously healthy man presented to our facility with a four-month history of progressive, unexplained systemic symptoms. His illness began with sudden, severe abdominal pain associated with elevated pancreatic enzymes and acute acalculus cholecystitis. Although inflammatory markers were markedly raised at that time, bacterial cultures were negative and no definitive infective source was identified. His symptoms improved only transiently following cholecystectomy and supportive care. Several weeks later, he developed recurrent episodes of chest pain, shortness of breath, and a brief loss of consciousness. Cardiopulmonary evaluation, including electrocardiography, cardiac biomarkers, and imaging including chest X-ray and echocardiogram (ECHO), did not reveal evidence of acute coronary syndrome, arrhythmia, pulmonary embolism, or pneumonia. He was again discharged after temporary symptomatic improvement. Over the subsequent two months, the patient experienced several episodes of bilateral lower limb swelling, erythema, and pain. These episodes were repeatedly diagnosed and treated as cellulitis in different hospitals. However, he reported that the affected areas became progressively dusky and violaceous (Figure 1) rather than erythematous, and he did not have persistent fever or purulent discharge. Antibiotic therapy produced minimal relief.

During this period, he also developed constitutional symptoms including fatigue, anorexia, and unintentional weight loss exceeding 4 kg. His blood pressure, previously normal, became intermittently elevated during symptomatic flares. Two weeks prior to presentation to our facility, he had developed neuropathic symptoms characterized by burning, tingling, and numbness affecting both hands and feet, more pronounced on the left. He reported that exposure to warm water markedly worsened the burning sensation, suggesting small and medium fibre ischemic involvement typical of vasculitic neuropathy. These features were clinically consistent with mononeuritis multiplex.

On arrival to our unit, he had multiple painful, dusky violaceous plaques and necrotic vasculitic lesions on both lower legs as seen in Figure 1 above. The lesions were indurated and tender but lacked the warmth, fluctuation, or lymphangitic streaking typical of cellulitis. Neurological examination revealed patchy, asymmetric sensory deficits in a mononeuritis multiplex pattern, with preserved motor strength and normal deep tendon reflexes. Cardiovascular and respiratory examinations were unremarkable. Notably, his dyspnea and myalgias improved dramatically within 24 hours of initiating systemic corticosteroid therapy.

Initial laboratory evaluation (Table 1) demonstrated a markedly inflammatory profile. C-reactive protein (CRP) was elevated and showed a steady decline with corticosteroid therapy, trending down at discharge and reaching a normal level at follow-up. The erythrocyte sedimentation rate (ESR) was elevated. Complete blood count revealed neutrophilic leukocytosis with elevated white blood cell count and absolute neutrophil count, which increased following steroid initiation, in keeping with a steroid effect. Hemoglobin was reduced at presentation with associated microcytosis and subsequently trended upward with treatment. Platelet counts were persistently elevated, consistent with reactive thrombocytosis. Ferritin was elevated.

**Table 1:** Summary of Laboratory Investigations During Hospital Course Laboratory findings in a patient with polyarteritis nodosa showing elevated inflammatory markers with progressive normalization following treatment and negative immunological and infectious workup. ANA, antinuclear antibody; ENA, extractable nuclear antigen; C-ANCA, cytoplasmic anti-neutrophil cytoplasmic antibodies PR3, proteinase-3; P-ANCA, perinuclear anti-neutrophil cytoplasmic antibodies; MPO, myeloperoxidase; HBsAg, hepatitis B surface antigen; HCV, hepatitis C virus; AST, aspartate aminotransferase; ALT, alanine aminotransferase

Parameter	Result / Trend	Units	Reference Range
C-reactive protein (CRP)	66 → 60 → 45 → 36 → 19 → 1.6	mg/L	<5
Erythrocyte sedimentation rate (ESR)	66	mm/hr	<20
Hemoglobin	9.7 → 11.2	g/dL	13.0–17.0
Mean corpuscular volume (MCV)	81.6	fL	83–101
White blood cell count	12.79	×10⁹/L	4.0–11.0
Absolute neutrophil count	9.27 → 11.01	×10⁹/L	2.0–7.5
Lymphocyte count	14	×10⁹/L	1.0–3.0
Platelet count	463–478	×10⁹/L	150–450
Ferritin	464	ng/mL	30–400
Fibrinogen	3.7	g/L	1.8–3.6
Complement C3	Elevated (2.7)	g/L	0.9–1.8
Complement C4	Normal	g/L	0.1–0.4
ANA	Negative	-	Negative
ENA panel	Negative	-	Negative
Anti-dsDNA	Negative	IU/mL	Negative
C-ANCA (PR3)	Negative	-	Negative
P-ANCA (MPO)	Negative	-	Negative
Cryoglobulins	Negative	-	Negative
HBsAg	Negative	-	Negative
Anti-HCV	Negative	-	Negative
HIV serology	Negative	-	Negative
Serum creatinine	64	µmol/L	64–104
AST	24	U/L	<22
ALT	34	U/L	<41

Renal and hepatic function tests were within normal limits. Electrolytes, lactate dehydrogenase (LDH), creatine kinase (CK), and lipid profile were normal. Complement C3 was elevated with a normal C4, suggesting an acute-phase response rather than hypocomplementemic immune-complex disease. Autoimmune serology revealed negative antinuclear antibody (ANA), extractable nuclear antigen (ENA) panel, and anti-double-stranded DNA antibodies. Anti-neutrophil cytoplasmic antibodies were negative for both cytoplasmic (C-ANCA) and perinuclear (P-ANCA) patterns. Screening for hepatitis B surface antigen, hepatitis C antibody, HIV, and cryoglobulins was negative.

**Figure 2:**
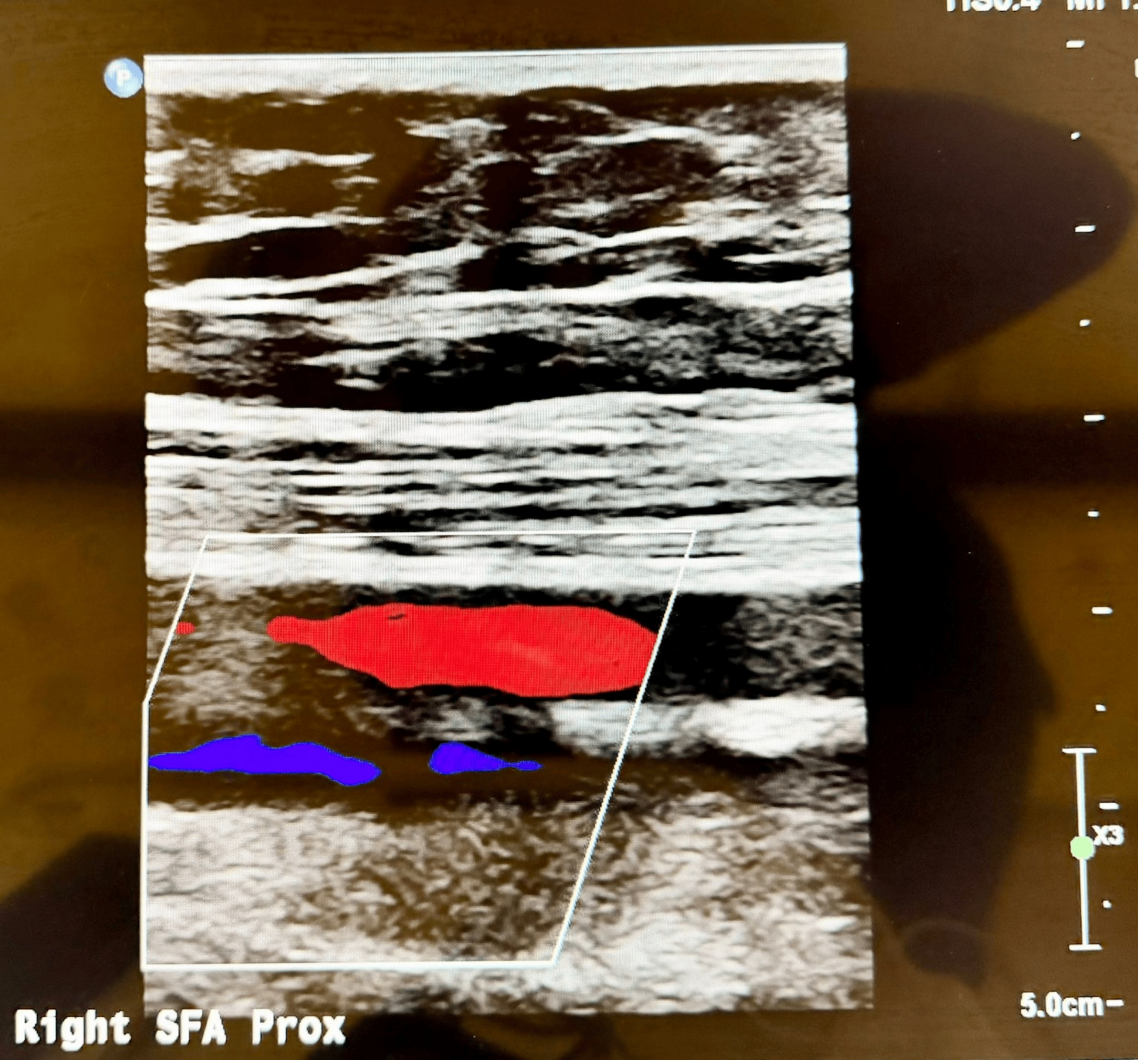
Normal Peripheral Arterial Ultrasound Grayscale and colour Doppler ultrasound demonstrating normal peripheral anatomy with preserved vessel caliber, intact wall morphology, and normal laminar blood flow. No aneurysmal dilatation, stenosis,or intraluminal thrombus was identified.

**Figure 3:**
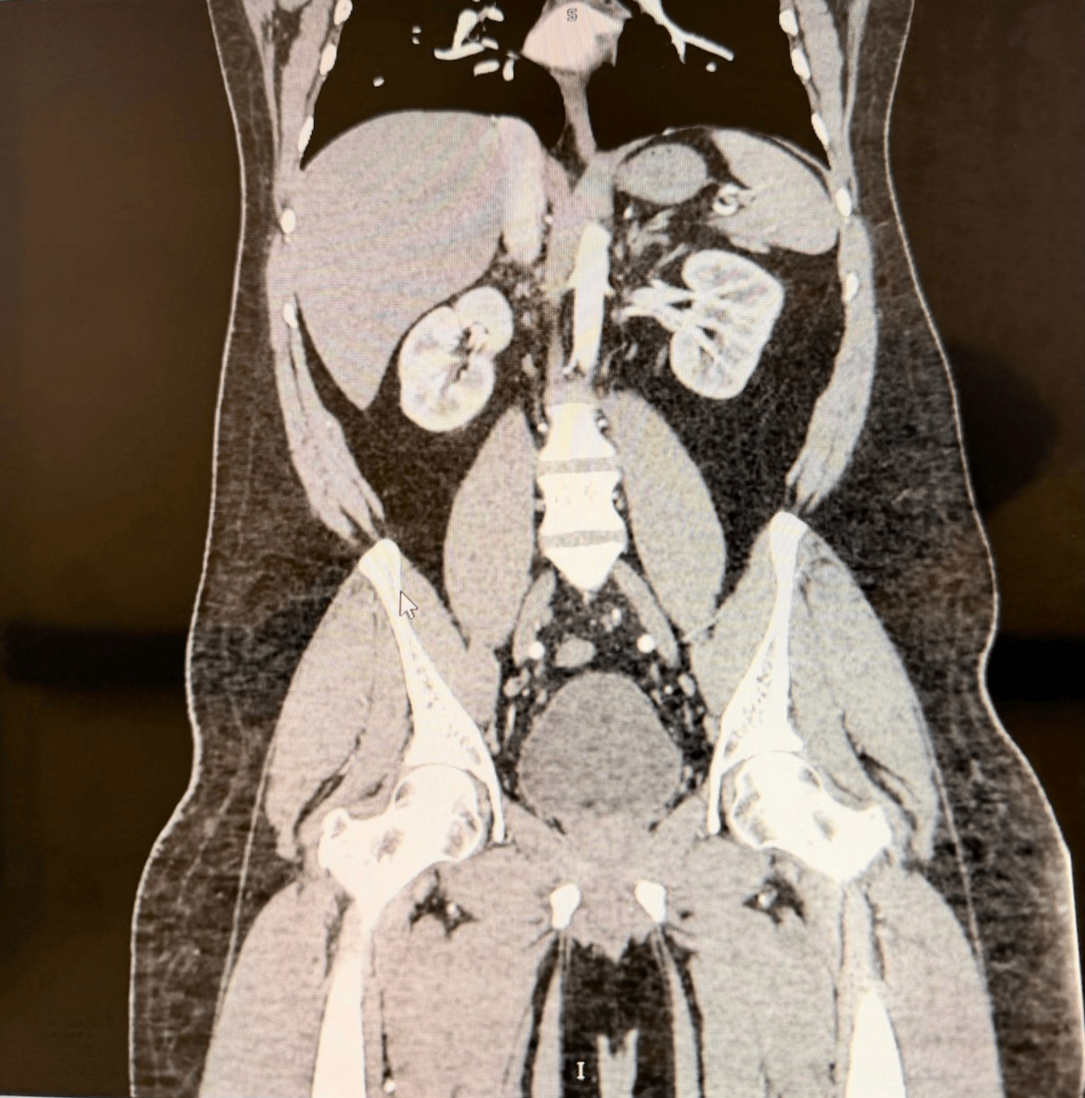
Normal CT Angiography of Abdomen and Pelvis Contrast-enhanced CT angiography of the abdomen and pelvis demonstrating normal abdominal vasculature with preserved vessel caliber and enhancement, without evidence of aneurysmal dilatation, stenosis,dissection or mural irregularity.

Arterial Doppler ultrasound of the lower limbs (Figure 2) demonstrated normal triphasic flow without evidence of stenosis or occlusion. CT angiography of the abdomen and pelvis (Figure 3) showed no aneurysms, stenoses, or beading of the renal, mesenteric, or other medium-sized arteries. These findings do not exclude PAN, especially in early disease when structural vascular changes have not yet developed.

**Figure 4:**
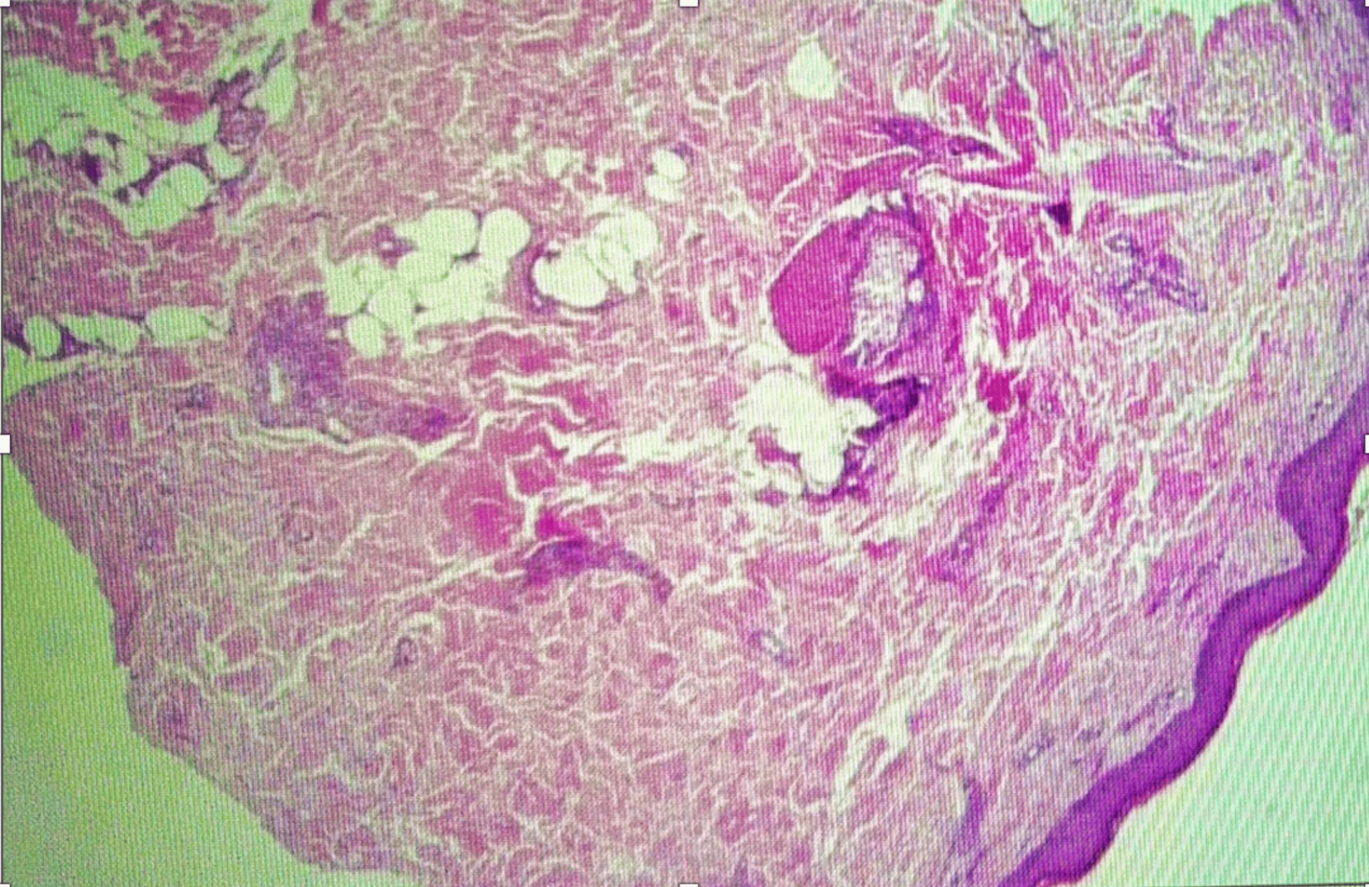
Skin Biopsy Demonstrating Superficial Perivascular Inflammatory Infiltrates

**Figure 5:**
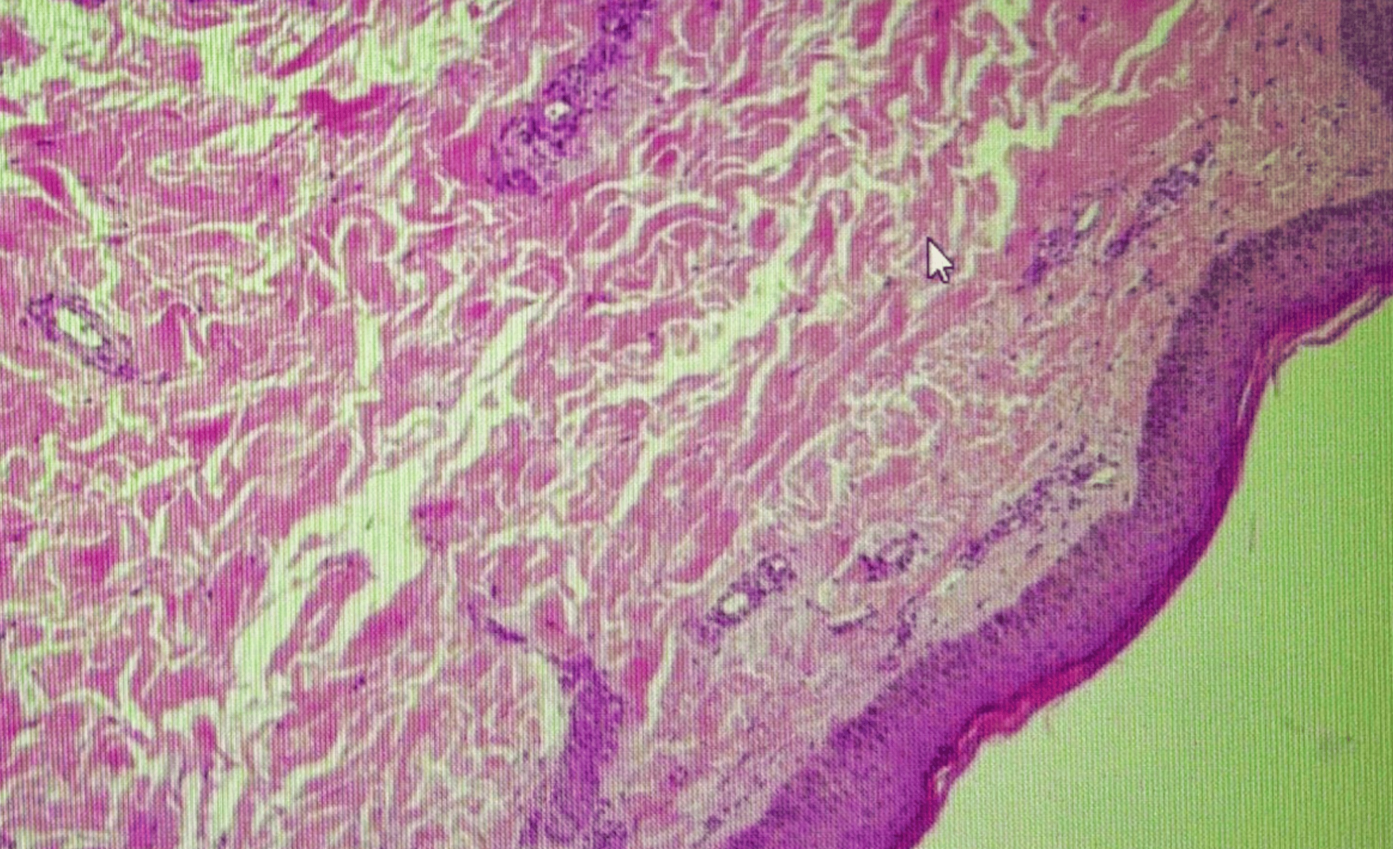
Histopathological Findings From Lower Leg Skin Biopsy

A punch biopsy was obtained from an older lesion on the lower leg after systemic corticosteroids had already been initiated. Histopathology revealed superficial and mid-dermal perivascular lymphocytic infiltrates with occasional eosinophils and minimal red cell extravasation. There was no fibrinoid necrosis or clear evidence of leukocytoclastic vasculitis. Importantly, the specimen did not include subcutaneous fat, and no medium-sized muscular arteries were present in the sections examined. The pathologist concluded that the findings were consistent with a dermal hypersensitivity reaction and stated that early vasculitis could not be excluded. Overall, the biopsy was considered nondiagnostic due to superficial sampling and post-steroid timing. Figure 4 and Figure 5 below show the biopsy results.

The working diagnosis at this stage was systemic necrotizing medium-vessel vasculitis clinically most consistent with PAN. High-dose intravenous dexamethasone (6 mg twice daily) was commenced and later transitioned to oral prednisolone 60 mg daily. Gabapentin was initiated and titrated for neuropathic pain. The patient’s skin tenderness, myalgias, and dyspnea improved markedly within days, and his neuropathic burning pain decreased although it did not completely resolve, as expected given the slower course of axonal recovery. Blood pressure stabilised, and inflammatory markers continued to fall. He was discharged on a tapering course of oral steroids and neuropathic pain medication with outpatient rheumatology follow-up.

## Discussion

PAN remains one of the most diagnostically challenging vasculitides because it lacks a single definitive laboratory or imaging marker and relies heavily on clinical synthesis. The 2012 Revised CHCC definition describes PAN as necrotizing inflammation of medium or small arteries without glomerulonephritis, arteriolar, capillary, or venular involvement and not associated with ANCA [2]. Our patient’s presentation aligns closely with this framework. He had no evidence of glomerulonephritis or pulmonary capillaritis; ANCA testing was negative; and the dominant features were medium-vessel-pattern neuropathy, necrotizing skin lesions, constitutional symptoms, and elevated acute-phase reactants.

The American College of Rheumatology (ACR) 1990 criteria for PAN (Table 2) were developed for classification rather than strict diagnosis, yet they remain a useful clinical guide. Meeting three or more of the 10 criteria yields a sensitivity of approximately 82% and specificity of 87% for PAN [8]. In this case, the patient clearly fulfilled at least five: significant unintentional weight loss, myalgias, mono- or polyneuropathy in the form of mononeuritis multiplex, elevated diastolic blood pressure during flares, and characteristic skin lesions including painful dusky plaques and necrosis. Although he did not meet angiographic or biopsy criteria, this is not uncommon; studies have demonstrated that a substantial proportion of clinically convincing PAN cases lack histologic or angiographic confirmation, particularly early in the disease course [6,7].

**Table 2:** American College of Rheumatology (ACR) 1990 Criteria for Polyarteritis Nodosa (PAN) in this Patient. Applications of the 1990 ACR classification criteria for PAN in the patient. The table summarises each criterion, its clinical definition, and whether it was met in this case [8]. BP, blood pressure; BUN, blood urea nitrogen; HBsAg, hepatitis B surface antigen; HBV, hepatitis B virus; CTA, CT angiography

ACR Criterion	Description	Present in this patient?
Weight loss >4 kg	Unintentional weight loss during disease course	Yes
Livedo reticularis	Livedo or dusky mottled/necrotic skin lesions	Yes (dusky necrotic vasculitic plaques)
Testicular pain/tenderness	Orchitis or testicular ischemia	No
Myalgias	Diffuse muscle pain or limb tenderness	Yes
Mononeuropathy or polyneuropathy	Asymmetric sensorimotor neuropathy	Yes (mononeuritis multiplex)
Diastolic BP >90 mmHg	New-onset hypertension during disease	Yes
Elevated BUN/creatinine	Renal impairment not due to dehydration	No
Hepatitis B infection	HBsAg or HBV correlation	No (HBV negative)
Arteriographic abnormality	Aneurysms or stenoses of medium arteries	No (CTA normal, early disease)
Biopsy of medium artery	Necrotizing arteritis in medium-sized artery	No (biopsy superficial and nondiagnostic)

A crucial aspect of the differential diagnosis (Table 3) was distinguishing PAN from AAV and immune-complex vasculitides. AAV, encompassing GPA, MPA, and EGPA, typically presents with glomerulonephritis, pulmonary hemorrhage or parenchymal nodules, and ENT involvement, alongside proteinase-3 (PR3)- or myeloperoxidase (MPO)-ANCA positivity in most cases. Our patient had negative ANCA, normal renal function, no urinary abnormalities, no pulmonary infiltrates or hemoptysis, and no ENT manifestations like chronic or recurrent sinusitis, persistent nasal crusting, epistaxis, etc., making AAV highly unlikely [3,4]. Immune-complex-mediated vasculitides such as cryoglobulinemic vasculitis generally feature low complement levels, especially low C4, and are often associated with hepatitis C or B infection and renal involvement. In contrast, this patient had elevated C3 as an acute-phase reactant, normal C4, negative cryoglobulins, and preserved renal function, effectively excluding cryoglobulinemic and other hypocomplementemic vasculitides [5,9].

**Table 3:** Comparison of Polyarteritis Nodosa (PAN) and ANCA-Associated Vasculitis (AAV) ANCA, anti-neutrophil cytoplasmic antibodies; GPA, granulomatosis with polyangitis; MPA, microscopic polyangitis; EGPA, eosinophilic granulomatosis with polyangitis; PR3, proteinase-3; MPO, myeloperoxidase; HBV, hepatitis B virus [2-4]

Feature	PAN	AAV (GPA/MPA/EGPA)
ANCA	Usually negative	Usually positive (PR3- or MPO-ANCA)
Predominant vessel size	Medium-sized muscular arteries	Small vessels ± medium arteries
Glomerulonephritis (GN)	Absent; no GN	Common; necrotizing GN with crescents
Pulmonary involvement	Rare; lung sparing typical	Common; alveolar hemorrhage or nodules
ENT involvement	Uncommon	Typical in GPA/EGPA
Neuropathy	Very common mononeuritis multiplex	Common but often with other systemic features
Skin lesions	Nodules, livedo, necrosis, ulcers	Palpable purpura, infarcts, nodules
Complement levels	Normal or mildly elevated (acute-phase)	Often normal; hypocomplementemia less typical
Biopsy findings	Necrotizing arteritis of medium arteries	Leukocytoclastic vasculitis ± granulomas
Angiography	Microaneurysms, stenoses in late disease	Often normal
Association with HBV	Classically associated, now less common	No strong association

Cutaneous PAN confined to the skin and superficial nerves can present with nodules, livedo, and neuropathy but tends to lack the systemic constitutional features and multisystem inflammatory episodes seen in this patient. His abdominal pain episodes associated with raised inflammatory markers, chest pain, constitutional features, and laboratory profile all support systemic PAN rather than a purely cutaneous variant.

The limitations of biopsy and angiography in this case mirror those described in major PAN series. Medium-sized arteries are located in the deep dermis and subcutis, and diagnostic yield is highest when deep incisional biopsies are obtained from early, active, tender lesions. Superficial punch biopsies that do not capture subcutaneous fat have poor sensitivity, often revealing only non-specific perivascular inflammation. Moreover, corticosteroids rapidly reduce inflammatory infiltrates, and biopsies performed even a few days after treatment begins may fail to show vasculitis. Similarly, CT angiography has limited sensitivity early in disease because microaneurysms, stenoses, and beading may take time to develop. Thus, a normal study does not exclude PAN in patients with compatible clinical features [7].

The therapeutic response in this case provides further support for PAN. The patient’s rapid improvement in dyspnea, myalgias, and skin tenderness, the progressive fall in CRP from 66 to 1.6 mg/L, and stabilisation of hemoglobin and platelets are all characteristic of treatment-responsive necrotizing vasculitis [8,10]. Neuropathic symptoms improved more slowly, consistent with the expected time course of axonal recovery following ischemic injury. Such a pattern is less typical of purely infectious or degenerative processes and strongly supports an immune-mediated vasculitic mechanism.

**Figure 6:**
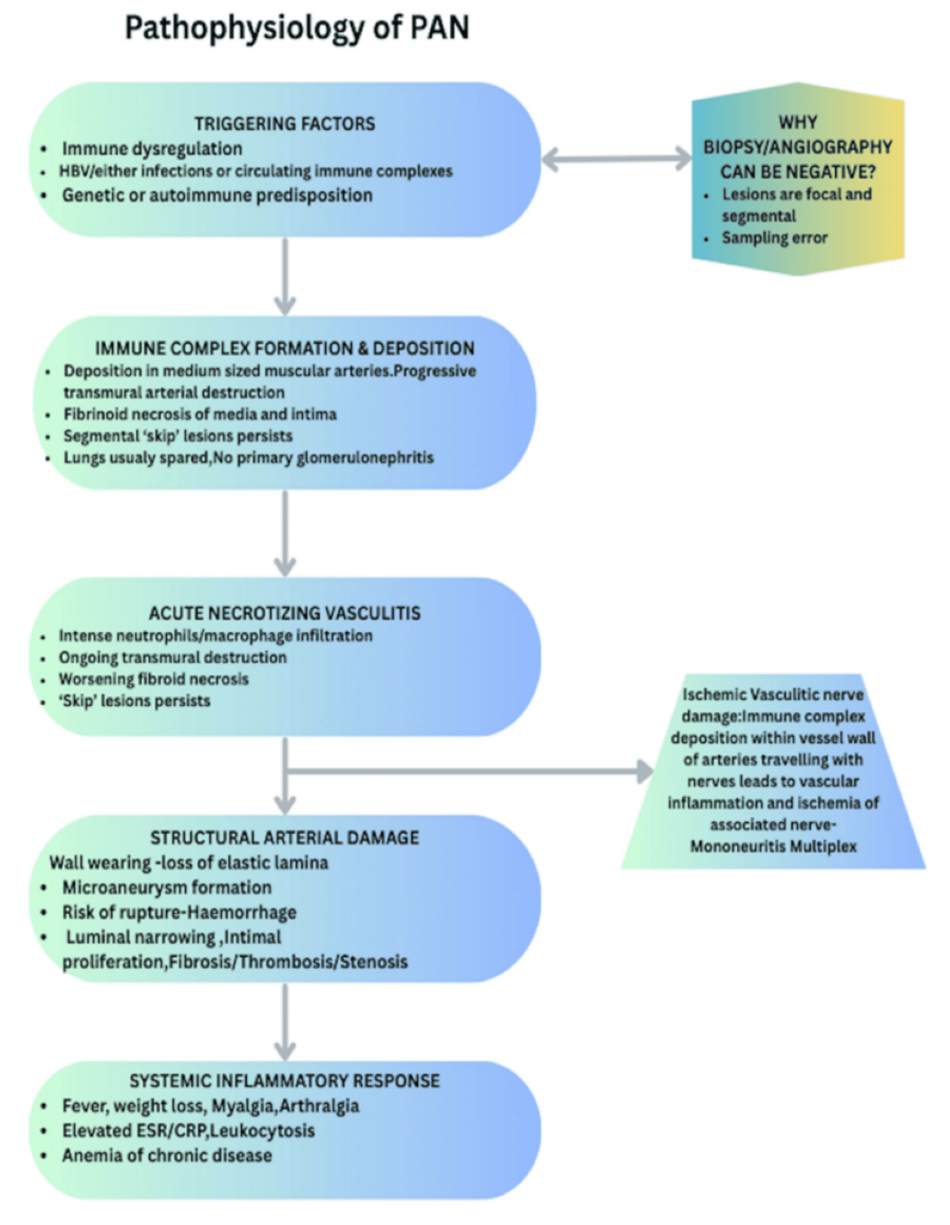
Pathophysiology of Polyarteritis Nodosa (PAN) Schematic overview of the pathophysiology of polyarteritis nodosa. The diagram illustrates proposed triggering factors, immune complex-mediated inflammation of medium-sized muscular arteries, progression to necrotising vasculitis, and resulting structural arterial damage leading to ischemic organ injury and systemic inflammatory manifestations, which need not happen in the same depicted order, and could have overlaps [10,11]. CRP, C-reactive protein; ESR, erythrocyte sedimentation rate; HBV, hepatitis B virus. Original figure created by authors.

Taken together, the multisystem involvement, mononeuritis multiplex, classical necrotizing skin lesions, high inflammatory markers, ANCA negativity, complement pattern, exclusion of other vasculitides and infections, and dramatic steroid responsiveness form a coherent picture of systemic necrotizing medium-vessel vasculitis clinically consistent with PAN (Figure 6). This case reinforces the concept that, in real-world practice, PAN frequently remains a clinical diagnosis where incomplete or nondiagnostic histology and imaging should not delay life-saving treatment [6,8,10].

Learning pearls

Early CT angiography can be normal in PAN; absence of aneurysms does not exclude disease. Superficial punch biopsies often miss medium-sized arteries and deep incisional biopsy is preferred. ANCA-negativity and absence of glomerulonephritis strongly favour PAN over AAV. Rapid steroid responsiveness is a key diagnostic and therapeutic clue in PAN. Mononeuritis multiplex is one of the most specific clinical features of medium-vessel vasculitis. PAN remains primarily a clinical diagnosis; normal imaging and nondiagnostic biopsy should not delay treatment. Complement pattern (normal C4, elevated C3) supports acute-phase response rather than immune-complex vasculitis. Early aggressive steroid therapy prevents irreversible nerve damage and organ ischemia.

## Conclusions

This case demonstrates a classical presentation of systemic necrotizing medium-vessel vasculitis clinically consistent with PAN in a young adult. Despite a superficial nondiagnostic skin biopsy and normal early CT angiography, the patient fulfilled multiple ACR criteria and exhibited multisystem involvement, mononeuritis multiplex, high inflammatory markers, and a marked steroid response. The case highlights the limitations of superficial biopsies and the low early sensitivity of angiography in PAN, emphasizing that clinical judgment remains central to diagnosis. Early recognition and prompt initiation of corticosteroids were crucial in preventing further organ damage and achieving significant clinical and biochemical improvement.
